# The Role of Spinal Cord Stimulation in Axial Back Pain

**DOI:** 10.7759/cureus.21980

**Published:** 2022-02-07

**Authors:** Colin Mychak, Shravan Gupta, Joseph E Mouhanna

**Affiliations:** 1 Pain Management, Larkin Community Hospital, Miami, USA

**Keywords:** opioid alternatives, neuromodulation, axial back pain, burst dr, spinal cord stimulation

## Abstract

Chronic axial low back pain (LBP) is one of the most common ailments in the United States, with a significant impact on quality of life and function. Multimodal therapy is often utilized for pain relief, including opioid pain medication. Current indications for spinal cord stimulation include chronic neuropathic conditions, such as failed back surgery syndrome, radiculopathies, complex regional pain syndrome types I and II, postherpetic neuralgia, and peripheral diabetic neuropathies. While current lead placements perform remarkably when used for their appropriate indications, there is no specific indication for spinal cord stimulation in the treatment of axial LBP. However, spinal cord stimulation lead placement at the T6 mid-vertebral body can be considered in patients with significant or predominant complaints of axial LBP. Achievement of pain relief via spinal cord stimulation can reduce the administration of both opioid and non-opioid pain medications.

## Introduction

Chronic pain, especially low back pain (LBP), is one of the leading causes of physical activity limitation, absence from work, and disability globally. The prevalence of LBP in 2017 was estimated to be 7.5% globally or approximately 577 million people [1], with the lifetime prevalence approaching 60-70% [2]. In the United States alone, the economic impact of workdays lost secondary to LBP has been estimated at 100-200 billion USD each year [2].

Neuromodulation has been used in medicine for over 50 years, with the first dorsal column stimulator implant performed in 1967 by Dr. Norman Shealy. The first spinal cord stimulators (SCS) were designed based on the gate control theory initially proposed by Melzack and Wall in 1965 [1,3]. They postulated that increased stimulation of non-nociceptive fibers of the dorsal columns would reduce or block signal transduction of smaller nociceptive fibers to supraspinal targets. Although there have been considerable improvements to the technology used in SCS today compared to those used by Dr. Shealy, they continue to rely on this theory.

Currently, Food and Drug Administration (FDA)-approved indications for spinal cord stimulation include failed back surgery syndrome or postlaminectomy pain, cervical and lumbar radiculopathy, complex regional pain syndrome types I and II, diabetic peripheral neuropathy, arachnoiditis, epidural fibrosis, post-thoracotomy pain, and postherpetic neuralgia. Currently, predominantly axial back pain in patients without a history of spine surgery is not an FDA-approved indication for SCS.

## Case presentation

A 53-year-old-female with a medical history of diabetes mellitus type 2, LBP, and lumbar radiculopathy presented to the clinic in December 2019 for LBP with intermittent radiation to the left lateral thigh. The patient reported progressively worsening pain secondary to a motor vehicle accident in 2016.

Initially, she responded well to medical management, and the radicular component of the pain was controlled. However, regarding the LBP, the patient failed multiple modalities, including physical therapy with home exercises, meloxicam, tizanidine, naproxen, and acetaminophen with codeine.

**Figure 1:**
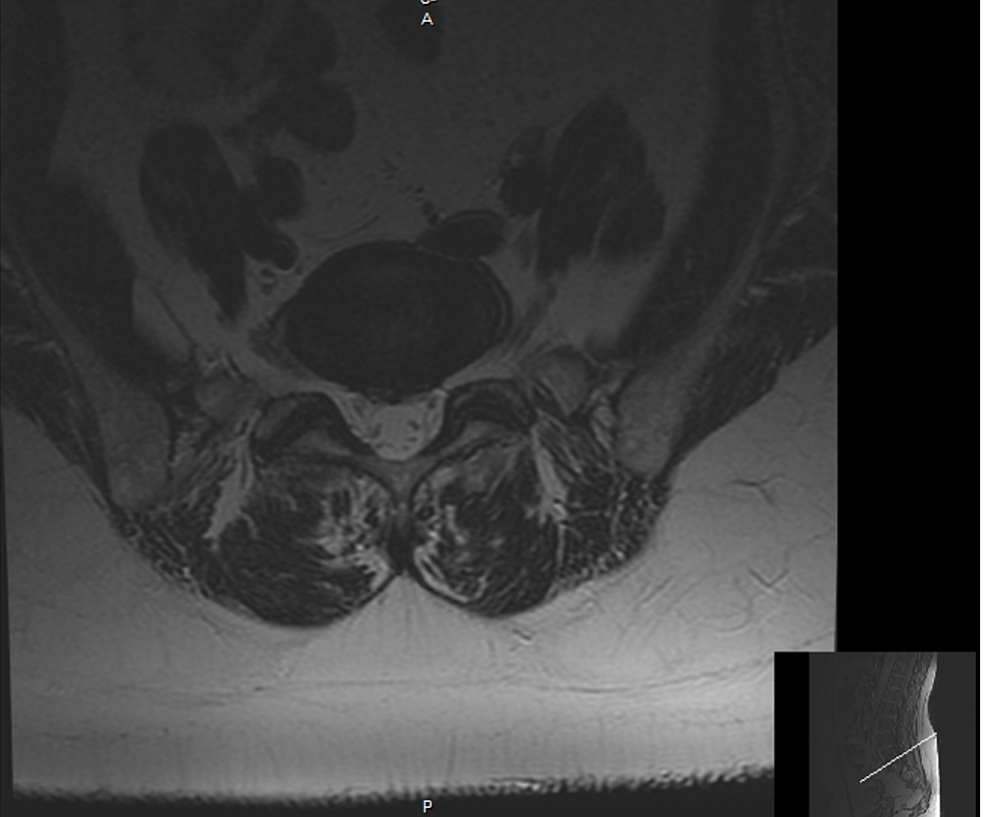
Lumbar MRI without contrast: axial view. MRI: magnetic resonance imaging

**Figure 2:**
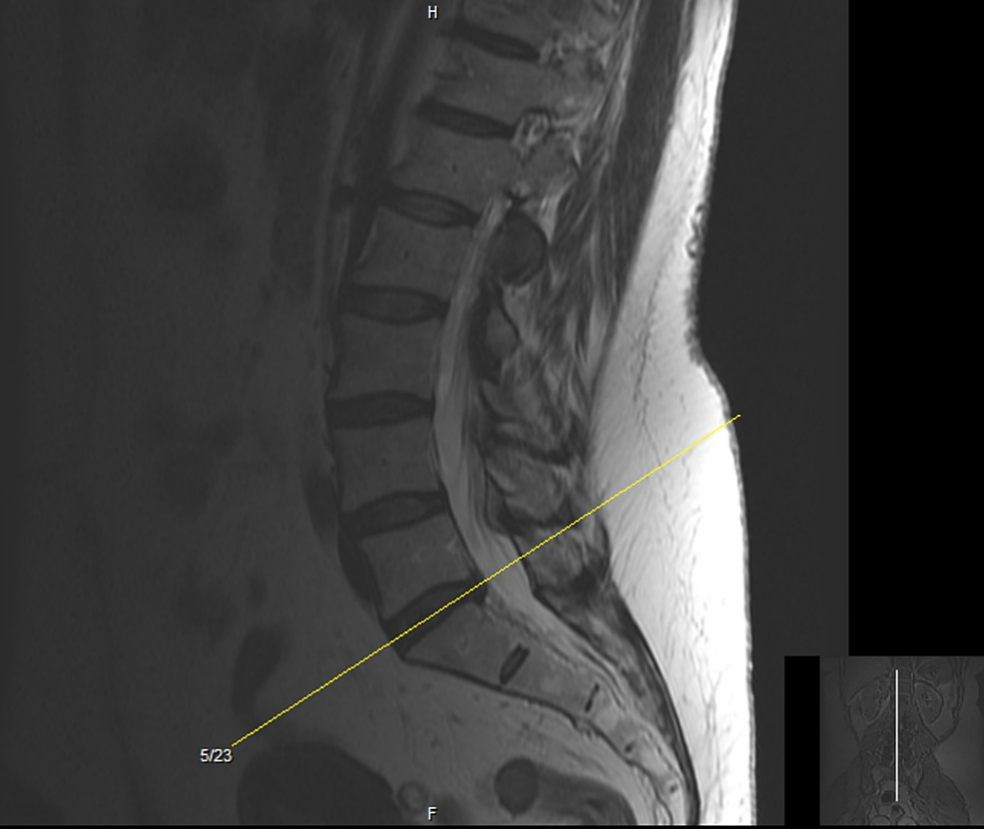
Lumbar MRI without contrast: sagittal view. MRI: magnetic resonance imaging

Lumbar magnetic resonance imaging (MRI) without contrast demonstrated chronic mild L1 compression fracture with 20% vertebral height loss. L4-L5 demonstrated mild global disc bulge and bilateral facet arthrosis, resulting in moderate bilateral neuroforaminal stenosis with mass effect on the exiting L4 nerve roots. L5-S1 demonstrated mild global disc bulge with shallow central disc herniation and bilateral facet arthrosis, resulting in minimal bilateral neuroforaminal stenosis (Figures 1, 2).

In December 2020, the patient underwent a series of diagnostic bilateral medial branch block and subsequent lumbar radiofrequency ablation to cover bilateral L4-L5 facets, which led to an 80% improvement in five months. In May 2021, the patient reported significant worsening of left lower extremity complaints and emergence of right lateral thigh pain. She was started on baclofen 10 mg BID, pregabalin 100 mg BID, and Percocet 5/325 mg BID with minimal relief. She received bilateral L4-L5 and L5-S1 transforaminal epidural steroid injections with significant but temporary relief. The patient then failed bilateral L5 neuroplasty. She noted severe reemergence of axial LBP in tandem with her radicular components. In November 2021, the decision was made to proceed with a trial of thoracolumbar spinal cord stimulation. Top lead contacts at the mid-T6 were chosen as the target level based on Barolat mapping. Two St. Jude/Abbott 8 contact leads were successfully introduced into the epidural space with Abbott epidural cannulas at the L1-L2 positioned at the mid-T6 vertebral body. Appropriate stimulation was confirmed in the area of the low back and bilateral lower extremities. Abbott’s Burst DR waveform was used at 0.5 mA. On follow-up three days later, the patient reported total resolution of her radicular pain, as well as 85% resolution of her axial LBP. She noted that she had not taken Percocet or baclofen the past two days due to the extent of her pain relief. At the time of this publication, the patient is awaiting placement of a permanent SCS implant and will continue on medication management.

## Discussion

Chronic axial LBP can be challenging to treat in many patients. In many cases, it is not well localized and can be the result of several independent pain generators. Although a thorough physical examination can oftentimes help identify these pain generators, it is not uncommon for a patient to have several pain generators contributing to the overall LBP syndrome. Innervation of a vertebral segment is intricate and involves sensory branches that supply both the osseous component as well as the intervertebral disc. The gray rami communicans, sinuvertebral nerve, basivertebral nerve, as well as medial, intermediate, and lateral branches of the dorsal rami are involved in the pain sensation of the spine [4]. However, of these, only the medial branches that innervate the facet joint and recently the basivertebral nerve, which provides innervation to the vertebral body, have been the target for intervention. While imaging studies can be useful in identifying a lesion responsible for a patient’s radicular pain, they offer little insight into the source of axial LBP. While in many cases there is evidence of diffuse spondylosis, disc degeneration, and facet arthropathy, these imaging findings may not correlate to physical examination findings [4]. Even when the imaging studies and the clinical picture align, limited therapeutic techniques exist currently for addressing axial LBP. This can be a cause for much frustration not only for the patient but also for the treating clinician. New novel treatments are currently starting to become available for some etiologies of LBP such as radiofrequency ablation of the basivertebral nerves. However there is no data yet to evaluate the long-term efficacy of these treatments.

Neuromodulation is an ever-evolving field that has gained significant attention in the past decade. As the technology and evidence base behind neuromodulation has developed, an increasing number of patients have become candidates for SCS. Current-generation SCS have smaller and longer-lasting battery life, can be programmed wirelessly, have improved MRI compatibility, and utilize novel stimulation waveforms. While traditional tonic SCS waveforms had limited ability to target axial LBP, newer sub-paresthesia waveforms, such as burst and high-frequency stimulation, have shown advancements in this area [3,5]. Recent studies have demonstrated the efficacy of these novel waveforms. In 2015, SENZA-RCT demonstrated the superiority of 10-kHz high-frequency therapy to traditional low-frequency SCS [6]. In 2018, the SUNBURST crossover trials demonstrated that patients preferred burst stimulation over low-frequency tonic stimulation. It also demonstrated superiority compared to traditional stimulation [7]. More recently, in 2020, a systematic review of 17 independent studies showed low-quality evidence that SCS can be utilized effectively to treat axial LBP [8]. What these studies lack is a specific target in the spinal cord for optimal coverage of axial LBP. In our patient, lead placement at the mid-T6 vertebral level delivered coverage of both radicular and axial LBP. However, a great deal of research needs to be done in the field of neuromodulation not only in terms of future waveforms but also ideal targets for therapy. As the technology and evidence base behind neuromodulation grows, we foresee axial LBP as an indication for spinal cord stimulation.

## Conclusions

With the prevalence of LBP amid the opioid epidemic, SCS may be a viable nonopioid modality. SCS reduce pain via the sensory or subsensory stimulation of A-beta fibers, which, in turn, causes a reduction in A-delta and C-fibers’ transmission of nocioceptive signals. While additional mechanisms have been proposed, their contribution to the efficacy of SCS remains unclear. Currently, LBP is not an indication for spinal cord stimulation. However, concomitant LBP and radicular symptoms are commonly encountered clinically. SCS lead placement at the T6 vertebral body level can be considered in patients complaining of axial LBP. Our case depicts the successful use of SCS in alleviating axial chronic LBP nonresponsive to conservative treatment modalities.
